# Cross Platform Standardisation of an Experimental Pipeline for Use in the Identification of Dysregulated Human Circulating MiRNAs

**DOI:** 10.1371/journal.pone.0137389

**Published:** 2015-09-10

**Authors:** Helena Kelly, Tim Downing, Nina L. Tuite, Terry J. Smith, Michael J. Kerin, Róisín M. Dwyer, Eoin Clancy, Thomas Barry, Kate Reddington

**Affiliations:** 1 Nucleic Acid Diagnostics Research Laboratory (NADRL), Microbiology, School of Natural Sciences, National University of Ireland, Galway, Ireland; 2 School of Biotechnology, Dublin City University, Dublin, Ireland; 3 Molecular Diagnostics Research Group (MDRG), School of Natural Sciences, National University of Ireland, Galway, Ireland; 4 Biomedical Diagnostics Institute (BDI) Programme, National University of Ireland, Galway, Ireland; 5 Discipline of Surgery, School of Medicine, National University of Ireland Galway, Galway, Ireland; National Institutes of Health, UNITED STATES

## Abstract

**Introduction:**

Micro RNAs (miRNAs) are a class of highly conserved small non-coding RNAs that play an important part in the post-transcriptional regulation of gene expression. A substantial number of miRNAs have been proposed as biomarkers for diseases. While reverse transcriptase Real-time PCR (RT-qPCR) is considered the gold standard for the evaluation and validation of miRNA biomarkers, small RNA sequencing is now routinely being adopted for the identification of dysregulated miRNAs. However, in many cases where putative miRNA biomarkers are identified using small RNA sequencing, they are not substantiated when RT-qPCR is used for validation. To date, there is a lack of consensus regarding optimal methodologies for miRNA detection, quantification and standardisation when different platform technologies are used.

**Materials and Methods:**

In this study we present an experimental pipeline that takes into consideration sample collection, processing, enrichment, and the subsequent comparative analysis of circulating small ribonucleic acids using small RNA sequencing and RT-qPCR.

**Results, Discussion, Conclusions:**

Initially, a panel of miRNAs dysregulated in circulating blood from breast cancer patients compared to healthy women were identified using small RNA sequencing. MiR-320a was identified as the most dysregulated miRNA between the two female cohorts. Total RNA and enriched small RNA populations (<30 bp) isolated from peripheral blood from the same female cohort samples were then tested for using a miR-320a RT-qPCR assay. When total RNA was analysed with this miR-320a RT-qPCR assay, a 2.3-fold decrease in expression levels was observed between blood samples from healthy controls and breast cancer patients. However, upon enrichment for the small RNA population and subsequent analysis of miR-320a using RT-qPCR, its dysregulation in breast cancer patients was more pronounced with an 8.89-fold decrease in miR-320a expression. We propose that the experimental pipeline outlined could serve as a robust approach for the identification and validation of small RNA biomarkers for disease.

## Introduction

Micro RNAs (miRNAs) are a class of highly conserved (18–25 nucleotide) small non-coding RNAs that play an important part in the post-transcriptional regulation of gene expression, making them essential to many fundamental pathological and biological processes [1,2] in all forms of life from animals [3], plants [4], even to some viruses [5]. MiRNAs have an important role in the manifestation of a wide range of diseases from autoimmune disorders to cancer [6–9].

The mode of action of miRNA is complex as multiple miRNAs may function together, either by synergy or in competition to target a single mRNA. Conversely, a single miRNA can moderate many targets [10,11]. It is estimated that miRNAs may be responsible for regulating up to 60% of eukaryotic mRNAs [12–14]. The ability to generate miRNA expression profiles is therefore helpful to better understand these processes.

Significant advances have been made in recent years in the field of miRNA analysis and the understanding of their importance to biological processes. These advances have demonstrated the potential utility of some of these miRNAs as biomarkers for disease. Specific miRNA signatures have been identified in tissues [15], circulating in the blood [16] and in cancers such as lung, ovarian and breast [17–19]. While many studies have identified specific miRNAs as candidate markers for disease, there are still a number of challenges to be overcome in order to demonstrate the clinical utility of these targets as true biomarkers of disease. Currently, there are three main methods employed to determine the global expression levels of miRNAs and to predict their potential as biomarkers for disease. These methodologies are: reverse transcription real-time PCR (RT-qPCR) [20,21], microarray [22–24], and small RNA sequencing [25,26].

While RT-qPCR is considered the gold standard for evaluation and validation of miRNA biomarkers [27,28], because it accurately quantifies low-copy miRNAs [29], small RNA sequencing is rapidly becoming routine for the initial identification of potential miRNA disease biomarkers. However, recent studies have demonstrated a lack of standardisation in the repertoire of small RNAs detected by high-throughput sequencing approaches [30,31]. In many cases where putative biomarkers are identified with small RNA sequencing, they are not substantiated when RT-qPCR is used for the validation of these novel miRNA biomarkers for disease [31–33]. Recent publications have highlighted a need for standardisation of upstream variables such as RNA sample preparation and downstream variables such as accurate quantification of enriched small RNAs [34]. Neither spiked-in RNA controls nor adjustment during bioinformatic processing are sufficient to account for technical artefacts and varying experimental protocols [35]. Moreover, measures of relative but not absolute RNA expression are comparable across platforms, but only if precise procedures are used [36]. These experimental design and normalisation strategies need to be addressed in order to overcome the limitations observed with small RNA sequencing for novel biomarker identification and subsequent validation of those putative biomarkers using RT-qPCR.

Recently, Redshaw et al., compared methods of small RNA isolation and enrichment, and subsequent quantitative analysis of purified miRNAs using two commercially available RT-qPCR kits. An important observation in this study demonstrated that when using RT-qPCR for determining miRNA absolute copy number in a sample, short RNA enrichment protocols (< 200 bases) can reduce the detected copy number of miRNAs by up to 25% when compared to using total RNA [33,34]. This demonstrates that it is not possible to accurately compare absolute miRNA copy number in enriched RNA populations to the total RNA population.

This observation may also have important implications when cross platform analysis between small RNA sequencing and RT-qPCR are performed. For example, prior to the generation of cDNA for deep sequencing, a gel purification step to enrich for small RNA (< 30) bases is typically performed [37]. This pre-enrichment step facilitates the removal of higher molecular weight RNAs including precursor miRNAs. Consequently, a purification step to enrich for small RNA of <30 bases may also be required for RT-qPCR to relate it to high-throughput sequence data. To our knowledge, there are currently no studies described in the literature evaluating this approach.

In this study we present an experimental pipeline for analysis of small RNA populations for both deep sequencing and RT-qPCR that facilitates reliable cross-platform analysis for the identification and validation of biomarkers associated with disease states. As a proof of concept for this approach, we identified a differentially expressed miRNA (miR-320a) in circulating blood among a cohort of healthy females (n = 23) compared to a cohort of female breast cancer patients (n = 14) based on Illumina sequencing data. We have further validated the miR-320a differential expression levels between these cohort groups using RT-qPCR when the experimental pipeline outlined in this study is adhered to.

## Materials and Methods

### Ethics Statement

Written informed consent was received from all participants in the study. Ethical approval was granted by the Clinical Research Ethics Committee, Galway University Hospital.

### Study Population

Peripheral blood samples were obtained from a total of 55 women, with 36 healthy female volunteers and 19 patients with a diagnosis of breast cancer (Table 1). The healthy blood sample donors were interviewed prior to sample collection to ensure there was no current illness or previous history of breast cancer. Small RNA sequencing was carried out on blood samples from 23 healthy controls and 14 breast cancer patients. These samples, together with a further independent cohort of blood samples from 13 healthy controls and 5 breast cancer patients, were used to validate the small RNA sequencing results using RT-qPCR analysis (n = 55 in total).

**Table 1:** Sample cohort set analysed using small RNA sequencing and RT-qPCR.

Sample Type	Age	Type^a^	Subtype^a^	Small RNA Sequencing	RT-qPCR
Cancer	48	Ductal	Luminal A	✓	✓
Cancer	85	Lobular	Luminal A	✓	✓
Cancer	83	Lobular	Luminal A	✓	✓
Cancer	54	Ductal	Luminal A	✓	✓
Cancer	81	Ductal	Luminal A	✓	✓
Cancer	71	Ductal	Luminal A	✓	✓
Cancer	47	Ductal	Luminal A	✓	✓
Cancer	48	Lobular	Luminal A	✓	✓
Cancer	84	Microinvasive	Luminal B	✓	✓
Cancer	65	Inflammatory	Her2	✓	✓
Cancer	52	Ductal	Luminal A	✓	✓
Cancer	60	Ductal	Luminal A	✓	✓
Cancer	57	Ductal	Luminal A	✓	✓
Cancer	68	Ductal	Luminal A	✓	✓
Cancer	81	Ductal	Luminal A	not performed	✓
Cancer	48	Lobular	Luminal A	not performed	✓
Cancer	64	Ductal	Luminal A	not performed	✓
Cancer	70	Ductal	Luminal A	not performed	✓
Cancer	84	Ductal	Luminal A	not performed	✓
Pre-Menopausal Healthy	32	NA	NA	✓	✓
Pre-Menopausal Healthy	26	NA	NA	✓	✓
Pre-Menopausal Healthy	25	NA	NA	✓	✓
Pre-Menopausal Healthy	27	NA	NA	✓	✓
Pre-Menopausal Healthy	24	NA	NA	✓	✓
Pre-Menopausal Healthy	32	NA	NA	✓	✓
Pre-Menopausal Healthy	30	NA	NA	✓	✓
Pre-Menopausal Healthy	30	NA	NA	✓	✓
Pre-Menopausal Healthy	30	NA	NA	✓	✓
Pre-Menopausal Healthy	26	NA	NA	✓	✓
Pre-Menopausal Healthy	30	NA	NA	✓	✓
Pre-Menopausal Healthy	26	NA	NA	✓	✓
Pre-Menopausal Healthy	28	NA	NA	✓	✓
Pre-Menopausal Healthy	24	NA	NA	✓	✓
Pre-Menopausal Healthy	30	NA	NA	✓	✓
Pre-Menopausal Healthy	30	NA	NA	✓	✓
Post-Menopausal Healthy	52	NA	NA	✓	✓
Post-Menopausal Healthy	57	NA	NA	✓	✓
Post-Menopausal Healthy	70	NA	NA	✓	✓
Post-Menopausal Healthy	62	NA	NA	✓	✓
Post-Menopausal Healthy	51	NA	NA	✓	✓
Post-Menopausal Healthy	56	NA	NA	✓	✓
Post-Menopausal Healthy	72	NA	NA	✓	✓
Pre-Menopausal Healthy	24	NA	NA	not performed	✓
Pre-Menopausal Healthy	30	NA	NA	not performed	✓
Pre-Menopausal Healthy	35	NA	NA	not performed	✓
Pre-Menopausal Healthy	23	NA	NA	not performed	✓
Pre-Menopausal Healthy	22	NA	NA	not performed	✓
Pre-Menopausal Healthy	30	NA	NA	not performed	✓
Pre-Menopausal Healthy	26	NA	NA	not performed	✓
Pre-Menopausal Healthy	30	NA	NA	not performed	✓
Post-Menopausal Healthy	53	NA	NA	not performed	✓
Post-Menopausal Healthy	64	NA	NA	not performed	✓
Post-Menopausal Healthy	54	NA	NA	not performed	✓
Post-Menopausal Healthy	59	NA	NA	not performed	✓
Post-Menopausal Healthy	52	NA	NA	not performed	✓

^**a**^ NA = Not applicable

### RNA Isolation and Purification

All whole blood samples for analysis in this study were collected in PAXgene blood tubes (Qiagen, Hilden, Germany). Total RNA, including small RNAs and miRNAs, were isolated and purified from 2.5ml of whole blood using the PAXgene blood miRNA kit (Qiagen, Hilden, Germany) per the manufacturer’s instructions [38]. Total purified RNA integrity was analyzed by capillary electrophoresis using a Bioanalyzer 2100 (Agilent Technologies, Santa Clara, California) and concentration and purity were measured by UV absorbance at 260nm using a NanoDrop 2000 (Thermo Scientific, Waltham, Massachusetts, USA). Isolated total RNA was stored at -80°C prior to use.

### Small RNA Sequencing

Small RNA library preparation and sequencing was carried out externally by BaseClear (Leiden, Holland) as described [39]. Briefly, small RNAs of lengths below 30 bases were enriched by polyacrylamide gel size selection purification. Illumina HiSeq adaptors were then ligated to the 5’ and 3’ ends of the small RNAs. These were amplified for 17 PCR cycles using primers for the adaptors. Post-amplification products were run on an agarose gel and the fragments of 90–110 bp, corresponding to the small RNA plus the adaptors, were isolated [40]. The purified retrieved PCR products were assessed for quality and quantity using the Agilent 2100 bioanalyser (Agilent Technologies). After determining quality and sufficient quantity, according to the manufacturer’s instructions, the library was sequenced on an Illumina HiSeq 2000 platform.

### Read Mapping and Annotation

The single-end 32 base RNA sequence reads generated were processed using the Illumina Casava pipeline tool (v1.8.3). Quality assessment was based on data passing the Illumina Chastity filtering [41]. Reads containing adaptors or PhiX control signal were removed. Quality assessment was based on the remaining reads using FASTQC v0.10.0 [42] to ensure base quality did not decline significantly across the read length. Small RNA 3' adapter (Illumina TruSeq) sequences and low quality bases were removed from the reads using the CLC Genomics Workbench v5.1.5 [43]. Reads smaller than 18 bases and those longer than 25 bases after trimming were excluded. Unique sequence groups were determined for each sample and these RNA sets were annotated as mature RNAs (IsomiRs) allowing for up to a two base variation in length up- or down-stream and up to one mismatch. The expression level of each small RNA was annotated using either miRBase *Homo sapiens*, or miRBase *Mus musculus*, or Ensembl *Homo sapiens* GRCh37 ncRNA database releases 57 and 67. Thus the data produced was analysed separately as a set of annotated miRNAs and a set of unknown small RNAs.

### Small RNA Sequencing Expression Normalisation and Testing

Differential RNA expression was evaluated for transcripts with a minimum of one count per million in at least three samples [44] using three normalisation methods: reads per kilobase per million mapped reads (RPKM) [45], trimmed mean of M-values (TMM) [46] and quantile normalisation [47]. Firstly, the fraction of reads mapping to small RNA or other small RNA transcripts for each sample was normalised within each sample as RPKM. Reads mapping to multiple RNAs were associated with those RNAs. Secondly, count data for each sample was normalised using quantile normalisation, arguably the optimal non-scaling normalisation approach with expression data aggregated in different sequencing runs [48]. This assumed the overall read count distributions were similar but can account for a large volume of zero or low, as well as high read counts [49]. Thirdly, the TMM value between sample pairs as a normalising factor reduced technical variation signals between small RNAs by compensating for differences in read levels between samples [46]. These were computed using R v2.15.0 Bioconductor package EdgeR v3.2.4 [50], which applied a negative binomial distribution model to identify differentially expressed miRNAs. Reads absent in one group but present in the other group required at least 10 reads in total to avoid sequencing artifacts. Multi-dimensional scaling (MDS) of the RPKM, quantile and TMM normalised levels within and between groups did not identify any samples as individual outliers. The miRNA and small RNAs examined for differential expression in this study had to satisfy all three RPKM, TMM and quantile normalisation methods using Welch’s t-test with Benjamini-Hochberg corrected two-tailed p-values < 0.05.

### Total RNA Standardisation for RT-qPCR

In order to ensure consistency in RNA from sample to sample, each total RNA sample was normalised to a concentration of 100 ng/μl based on UV absorbance at 260 nm (nanodrop).

### Small RNA Enrichment for RT-qPCR

Small molecular weight RNA enrichment (approximately 16 to 25 nucleotide bases) was achieved by a process of size selection using polyacrylamide gels. Fifteen μl of total RNA from each sample, at 100 ng/μl, was applied to a 15% polyacrylamide TBE urea gel. This RNA was electrophoresed alongside a size-specific RNA indicator consisting of RNA oligonucleotides (MWG-Biotech AG, Essenberg, Germany) of between 16 and 23 bases in size. These gels were stained using an ethidium bromide bath and imaged by UV transilluminator. The size markers were utilized to indicate the gel region corresponding to the small RNA of interest present in a sample. These indicated regions were then excised and the RNA retrieved, in an elution volume of 15μl of nuclease free water (Life technologies, Carlsbad, CA, USA), using a small RNA PAGE recovery kit (Zymo Research, Irvine, CA, USA). This process ensured that the volume was identical both pre- and post-enrichment and that the subsequent RT-qPCR reactions were carried out using the same effective volumes of total RNA and enriched small RNA [33].

In order to assess and control for the efficiency of the small RNA enrichment process an external spike-in normalisation control was required. A miRNA of *Arabidopsis* origin (Aly-miR-159) was chosen for this control and was spiked into all total RNA samples prior to size selection at a final concentration of 1.2 x 10^7^ copies per μl as previously described [33]. This miRNA was chosen as an external spike-in control due the lack of sequence homology to human miRNAs published on miRBase [33,51]. This spike in control has the potential to act as an appropriate control for human miRNA studies when added to a sample under investigation prior to purification of nucleic acid.

### MiRNA Reverse Transcription

Reverse transcription reactions were performed using the Taqman miRNA Reverse Transcription Kit (Life Technologies) and MMLV reverse transcriptase (Life Technologies) on RNA, both total and enriched, isolated from each individual clinical sample. The reaction mix for the reverse transcription process was comprised of 1μl of 10x first strand buffer, 1μl dNTP nucleotide mix, 2μl specific Taqman miRNA reverse transcription assay, 4.5μl nuclease free water and 0.5μl of MMLV reverse transcriptase per reaction. Template RNA (1μl) was then added. The thermocycling protocol used was as follows: 16°C for 30 min, 42°C for 30 min, 85°C for 5 min. Reverse transcription was carried out on the same volumes of enriched small RNA eluant and the corresponding non-enriched samples (total RNA). Reactions were performed on a thermocycler (PTC-200 MJ Research, Minnesota, USA).

All reactions were carried out in triplicate and appropriate controls were incorporated into each run: namely a no reverse transcriptase negative control (RT NEC) and a no template negative control (RT NTC). The produced cDNA was diluted using water (1:50) and stored at -20°C prior to qPCR analysis.

### Standard Curves and Quantitative Real Time-PCR

Absolute quantification was achieved for each specific miRNA by the generation of standard curves. Synthetic oligonucleotide representing the specific miRNA under investigation, both human and Arabidopsis, were synthesized by Integrated DNA technologies (Coralville, Iowa, USA). These were diluted to the appropriate concentration ranges and reverse transcribed as outlined above. All cDNA was diluted 1:50 and stored at -20°C prior to qPCR analysis. To control for run to run variation, internal standard curves were included in each qPCR experiment. A line was fit to the data points obtained for each standard dilution using the quantification cycle (Cq) values reported. This standard curve was then used to absolutely quantify the number of copies of the target miRNA present in the sample by comparing the Cq values observed for each sample to the relevant standard curve.

Quantitative PCR reactions were performed in accordance with the Minimum Information for Publication of Quantitative Real-Time PCR Experiments (MIQE) guidelines. All qPCR were performed on a LightCycler480 using the LightCycler480 probes master kit (Roche Diagnostics, Basel, Switzerland) and Taqman miRNA assays (Cat. No. 4427975, Life Technologies). All Taqman assays were available predesigned and purchased from Life Technologies with the exception of the aly-miR-159 assay which was custom designed and also purchased from Life Technologies. A final volume of 20μl was used for each reaction mixture. The optimized master mix contained 2× LightCycler 480 probes master (6.4 mM MgCl_2_), Taqman miRNA assay (1μl), template cDNA (1μl), spike-in control (1μl) and the volume was adjusted with the addition of nuclease-free dH_2_O.

The qPCR thermocycling conditions were as follows: 95°C for 10 min, followed by 50 cycles of 95°C for 15 s, 60°C for 1 min, followed by a cooling step at 40°C for 10 s. The temperature ramp rate on the LightCycler 480 instrument was 4.4°C/s while heating and 2.2°C/s while cooling. The LightCycler 480 Software, Version 1.5 (Roche) was used to calculate the quantification cycle (Cq) value. The qPCR runs were controlled using qPCR no template controls (RT NTC) in addition to the reverse transcription controls (RT NEC).

### Small RNA Enrichment Process Normalisation

The median quantification cycles observed in the control assay, specifically 27.4 (Total RNA) and 31.78 (enriched RNA) were used to calculate normalisation factors for the total RNA samples and enriched small RNA samples as previously described [52]. This normalisation factor was then used to control for any potential loss of RNA through the processing of the sample. This normalisation factor was applied to the RT-qPCR data obtained for all assays as outlined. The application of the normalisation factor allowed correction for any differences in recovery of RNA after small RNA enrichment, thus removing the potential for inaccurate quantification due to sample processing methods.

## Results

### Total RNA Isolation and Purification

All isolated total RNA samples were found to have an RNA integrity number (RIN) of 8 or greater. Based on the RIN numbers and quantities of total RNA obtained (Nanodrop), the extracted and purified RNA samples were determined to be of sufficient quality and quantity to enable processing for small RNA sequencing and RT-qPCR.

### Global Analysis of MiRNA from Healthy Controls and Breast Cancer Patients

Sequencing by synthesis amplification of the small RNA profile isolated from peripheral blood yielded 175,267 different miRNAs representing 1,211 known annotated miRNAs [53]. A further 138,457 were unannotated small RNAs and 9,227 were identified as putative tRNA fragments. No global difference was observed between the blood samples from healthy control group and breast cancer patients (S1 Fig) and unannotated small RNAs (S2 Fig). Both groups were dominated by miR-451a, which was the most highly expressed miRNA, accounting for 60% (healthy) and 65% (breast cancer) of the total. The next most frequently observed miRNAs were miR-191 (13% of control and 11% of breast cancer) and then miR-484 (3% and 4%).

### Differential Expression of Specific MiRNAs from Small RNA Sequencing Data

To test for differential expression of circulating miRNAs between the healthy control group and breast cancer patients, only miRNAs with adequate RPKM-scaled expression levels were examined to remove bias due to differences in sequencing depths. These miRNAs were normalised using both upper quantile and TMM methods: t-tests were performed such that differential expression was considered valid only if it was consistently different using all three (RPKM, quantile and TMM) methods after multiple test correction [50]. Interrogation of the miRNA results using RPKM adjustment alone would have yielded six differentially expressed miRNAs (miR-320a, miR-140, miR-30d, miR-22, miR-191, and miR-150). Of these potentially differentially expressed miRNAs, three (miR-191, miR-150 and miR-30d) were not supported after TMM testing, and only miR-320a and miR-140 were differentially expressed using upper quantile normalisation as well (Table 2).

**Table 2:** Differential expression patterns separating breast cancer patient from control samples.

miRNA	RPKM values				Upper quantile normalised				TMM normalised			
	t	p-value	Rank		t	p-value	Rank		t	p-value	Rank	
miR-320a	3.35	0.00187	5	*	5.1	0.00002	1	*	3.96	0.00032	1	**
miR-140	3.74	0.00068	3	*	3.35	0.00203	2	*	2.93	0.00585	2	**
let-7b	1.35	0.18649	10		3.22	0.003	3	*	2.78	0.00869	3	**
miR-30d	4.96	0.00001	1	*	2.2	0.03727	6		2.35	0.02555	6	
miR-150	2.95	0.00535	6	*	1.97	0.05679	7		0.52	0.60688	16	
miR-22	3.82	0.00048	2	*	1.71	0.09918	8		2.71	0.01072	4	**
miR-425	1.47	0.15133	9		1.05	0.30512	12		1.42	0.16665	12	
miR-191	3.54	0.00113	4	*	0.85	0.40409	14		1.39	0.17443	13	

Differential expression testing for eight miRNAs in breast cancer patients (15) and controls (26) that showed some evidence of differential expression across quantile, RPKM and TMM normalisation methods. Welch t-tests for differential expression using RPKM values, upper quantile normalised rates and TMM normalised levels all supported lower miR-320a and miR-140 expression in breast cancer patients. The two-tailed p-values were Benjamini-Hochberg corrected to adjust for multiple testing and those with p<0.05 are marked *.

**Fig 1:**
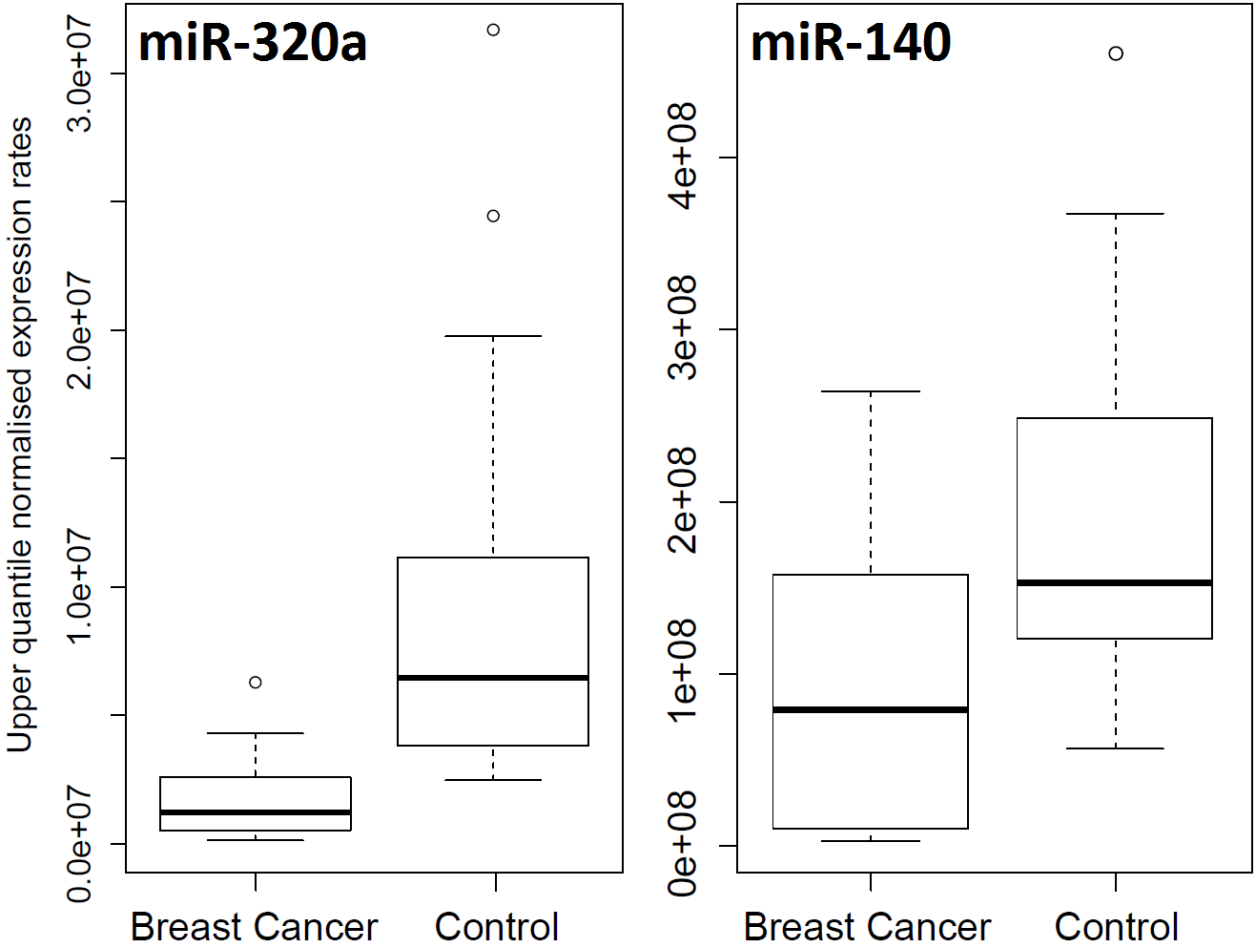
Differential analysis of miRNA between breast cancer and control groups. The distribution of miR-320a and miR-140 upper quantile normalised expression levels between the breast cancer and control groups from Illumina reads. Note that the y-axes are different for each miRNA because of the differing scales.

These two miRNAs showed lower expression in the blood samples from breast cancer patients relative to the healthy control group: miR-320a had the highest fold-change of 2.30, and miR-140 one of 1.72 (Table 3). Overall, the highest 15 miR-320a expression levels were observed in healthy female samples, while the lowest observable expression levels for miR-320a were associated with samples from breast cancer patients (Fig 1). The miR-320a effect size (Cohen’s d = 1.05) indicated a high probability (89%) of detecting a true differential expression (Table 3). The largest t-test p-value for miR-320a was 0.00187 (RPKM t = 3.35), and this was supported by the upper quantile (p = 0.00002) and TMM (p = 0.00032) normalised values. For these sequencing data, the vast majority of miR-320a isoforms detected were mature transcripts (99.6%) of which 83.1% were a single sequence (AAAAGCTGGGTTGAGAGGGCTAT). 13.8% of miR-320a sequences were identical to this sequence with an additional A nucleotide at the 3’ end. The remainder were rare isoforms.

**Table 3:** Micro RNA expression counts and variation between breast cancer patient and control samples.

miRNA	Control count		Breast cancer count		Fold change	Difference	Power
	Mean	SD	Mean	SD			
miR-320a	845	528	368	379	2.3	1.05	0.89
miR-140	3,687	1,407	2,141	1,190	1.72	1.19	0.95
let-7b	606	441	424	401	1.43	0.43	0.25
miR-30d	4,137	1,532	2,255	896	1.83	1.55	0.99
miR-150	2,176	1,092	1,388	617	1.57	0.92	0.79
miR-22	3,070	1,301	1,904	647	1.61	1.2	0.95
miR-425	2,485	892	2,087	807	1.19	0.47	0.29
miR-191	30,283	13,812	16,598	10,679	1.82	1.12	0.92

Expression values for eight miRNAs in breast cancer patients (15) and controls (26) that showed some evidence of differential expression. FC stands for the RPKM fold expression change (control/condition). Difference is Cohen’s d: the mean change in miRNA expression between groups scaled by the pooled SD (standard deviation), which reflects the effect size. Power indicates the probability of detecting true differential expression for that miRNA given the difference observed, alpha value (0.05) and sample sizes.

For miR-140, the largest t-test p was <0.006 (TMM t-test = 2.93), and the effect size (d = 1.19) suggested high power (95%) to find changes in expression if present (Table 3). RPKM and upper quantile normalised t-test p-values supported a lower miR-140 expression in the blood samples from breast cancer patients relative to healthy control group (p<0.001 and p = 0.002 respectively, Fig 1). Most miR-140 isoforms detected were mature transcripts (90.7%): 46.0% were a 23-base sequence (TACCACAGGGTAGAACCACGGAC) that differed by an additional 5’ T nucleotide and a substitution of C>A at site 14 compared to the second most abundant isoform (40.1%, ACCACAGGGTAGACCCACGGAA). The remainder were rare isoforms.

Let-7b showed slightly lower expression levels in the blood samples from breast cancer patients relative to the healthy control group–however, the smaller effect size (0.43) indicated that the limited sample size here resulted in low power (25%) to detect true expression differences if present. Further work involving sequencing at a higher read depth to mitigate the low let-7b expression levels should evaluate if the upper quantile (t = 3.22, p = 0.003) and TMM (t = 2.78, p<0.009) normalised tests are more accurate reflection of expression state differences than the RPKM results (t = 1.35, p = 0.187). The vast majority of let-7b isoforms detected were mature transcripts (95.6%): 73.6% were a single sequence (TGAGGTAGTAGGTTGTGTGAC). All other distinct isoforms were rare (<8%).

### Low Expression of Unannotated Small RNAs from Small RNA Sequencing Data

The small RNA profiles consisted of mainly miRNAs–the unannotated small RNAs fraction accounted for just 2.5% (healthy) and 2.6% (breast cancer) of the total. The distribution of expression values was more even than the annotated miRNAome: most highly expressed unannotated small RNA (ENST00000364228) represented 0.66% of the control and 0.93% of the breast cancer unannotated small RNAs. A total of 47 small unannotated RNAs were detected exclusively in the breast cancer samples with a total count level across samples >11. Five had expression levels greater than the mean total counts per gene in the cancer cohort (57.9, S1 Table)–the remaining 42 were rare. Of these five, ENST00000490626 was detected most frequently in the breast cancer cohort (7 out of 14). No small unannotated RNAs were exclusively differentially expressed across the healthy control samples. The data discussed in this publication have been deposited in NCBI's Gene Expression Omnibus [54] and are accessible through GEO Series accession number GSE72080 (http://www.ncbi.nlm.nih.gov/geo/query/acc.cgi?acc=GSE72080).

### MiRNA Target Selection and Small RNA Enrichment

MiR-320a was analysed further using RT-qPCR in an expanded sample set to examine the expression level of miR-320a. Let-7b was also analysed using RT-qPCR because it was previously reported to be differentially expressed between blood of breast cancer patients and healthy controls [14]. Similarly, miR-195 expression was assessed using RT-qPCR because it was dysregulated in breast cancer patients from the same geographical location as evaluated in this study [19]. MiR-140 was not evaluated using RT-qPCR because its ranked expression did not partition the groups as clearly as miR-320a. MiR-16 expression levels were analysed with RT-qPCR as a stable endogenous control [18].

**Fig 2:**
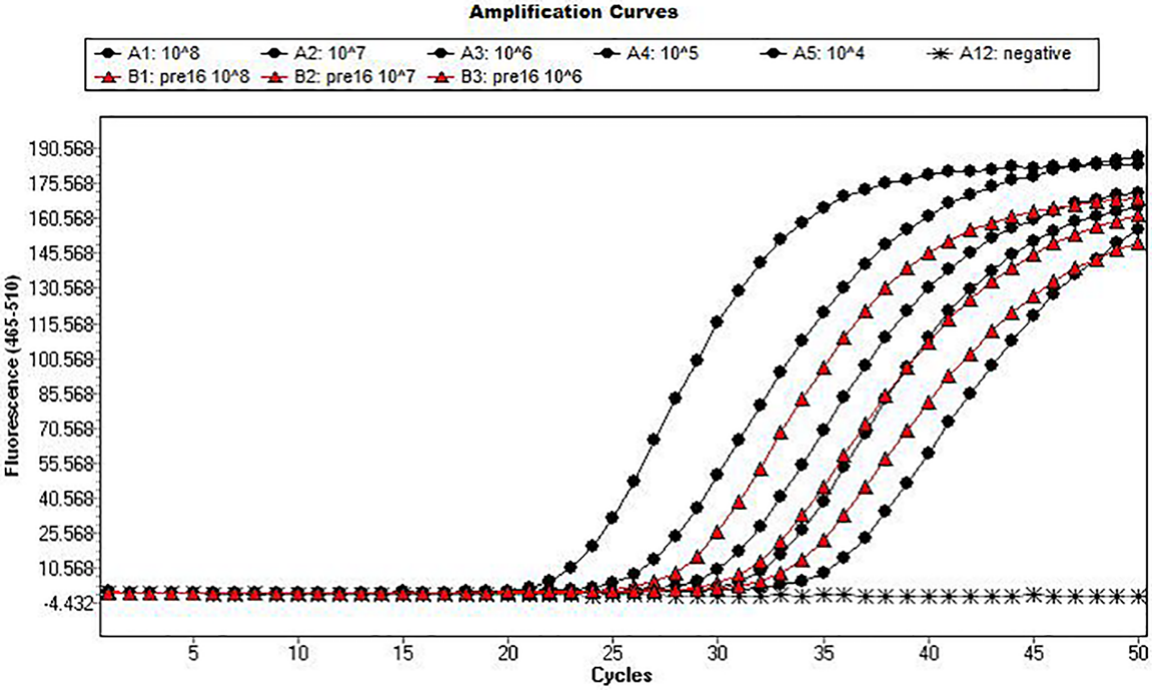
Analysis of precursor and mature miR-16 sequence using real-time PCR. Real-time PCR amplification curves detecting the mature miR16 standard curve 10*8–10*4 (circles- decreasing in concentration from left to right) with detection of precursor miR-16 sequence from 10*8–10*6 (triangles- decreasing in concentration from left to right) with the no template control, highlighted with diamonds in the FAM channel (465 to 510 nm).

Based on the sequence similarity between precursor and mature miRNAs, it was hypothesized that RT-qPCR assays may detect both precursor and mature sequences. The detection of the precursor would directly impact on the quantification of the absolute numbers of mature miRNAs. In order to demonstrate the potential for such an error in quantification through the use of total RNA in the measurement process, we included a synthetic miR-16 precursor sequence as a control template in the miR-16 assay. From this analysis we demonstrated that the miR-16 precursor sequence was also detected by the miRNA assay (Fig 2, S2 Table).

To reduce the potential for non-specific amplification and quantification of precursor sequences, a size selection small RNA enrichment process was carried out on all clinical samples to be analysed using RT-qPCR. This enrichment for RNA of approximately 30 bases or less resulted in a decrease in miRNA copy numbers detected by 4.1 to 11.7-fold (p<0.01, Table 4).

**Table 4:** Impact of small RNA enrichment on miRNA measurement.

Two-sample T-test	Mean	Stdev	SE Mean	Fold change Total to Small
				Decrease
Total miR-195	6.88E+07	8.34E+07	1.87E+07	
Enriched Small miR-195	5.87E+06	6.00E+06	1.38E+06	11.70
Total miR-16	2.52E+08	2.56E+08	5.72E+07	
Enriched Small miR-16	4.88E+07	4.96E+07	1.14E+07	5.20
Total miR-320a	1.30E+08	1.60E+08	3.58E+07	
Enriched Small miR-320a	3.16E+07	3.80E+07	8.95E+06	4.10
Total Let-7b	1.99E+07	2.66E+07	5.94E+06	
Enriched Small Let-7b	3.64E+06	4.83E+06	1.14E+06	5.50

### Standard Curve Generation and RT-qPCR

Absolute quantification was also achieved for each specific miRNA evaluated in this study by the generation of standard curves. Each miRNA target was represented by a standard curve of at least 6 sequential dilutions in order to allow for accurate quantification of the target miRNA in line with current best practices [55]. The standard curve for the miRNA target miR-195 and miR-16 ranged from 10^8^ to 10^3^ copies. The standard curve for the miRNA target miR-320a and Let-7b ranged from 10^9^ to 10^4^ copies. The standard curve for the spike-in control miRNA (Aly miR-159) ranged from 10^9^ to 10^4^ copies (S3 Fig). These standard curves were analyzed in each quantification experiment and demonstrated a high degree of consistency and accuracy with amplification efficiencies of between 1.8 and 2.1. MiR-320a, let-7b, miR-195 and miR-16 were analysed and quantified using RT-qPCR in samples from both the healthy controls and breast cancer patients using both total RNA and enriched small RNA. RT-qPCR quantification was also carried out for the spike-in control miRNA (aly-miR-159) in each sample analysed. The quantification cycle number measurement was consistent across the sample sets with a median of 27.4 and a mean of 28.3 observed for the spike-in control in the total RNA samples. A median of 31.78 and a mean of 31.8 was observed for the spike-in control in the enriched small RNA samples.

**Fig 3:**
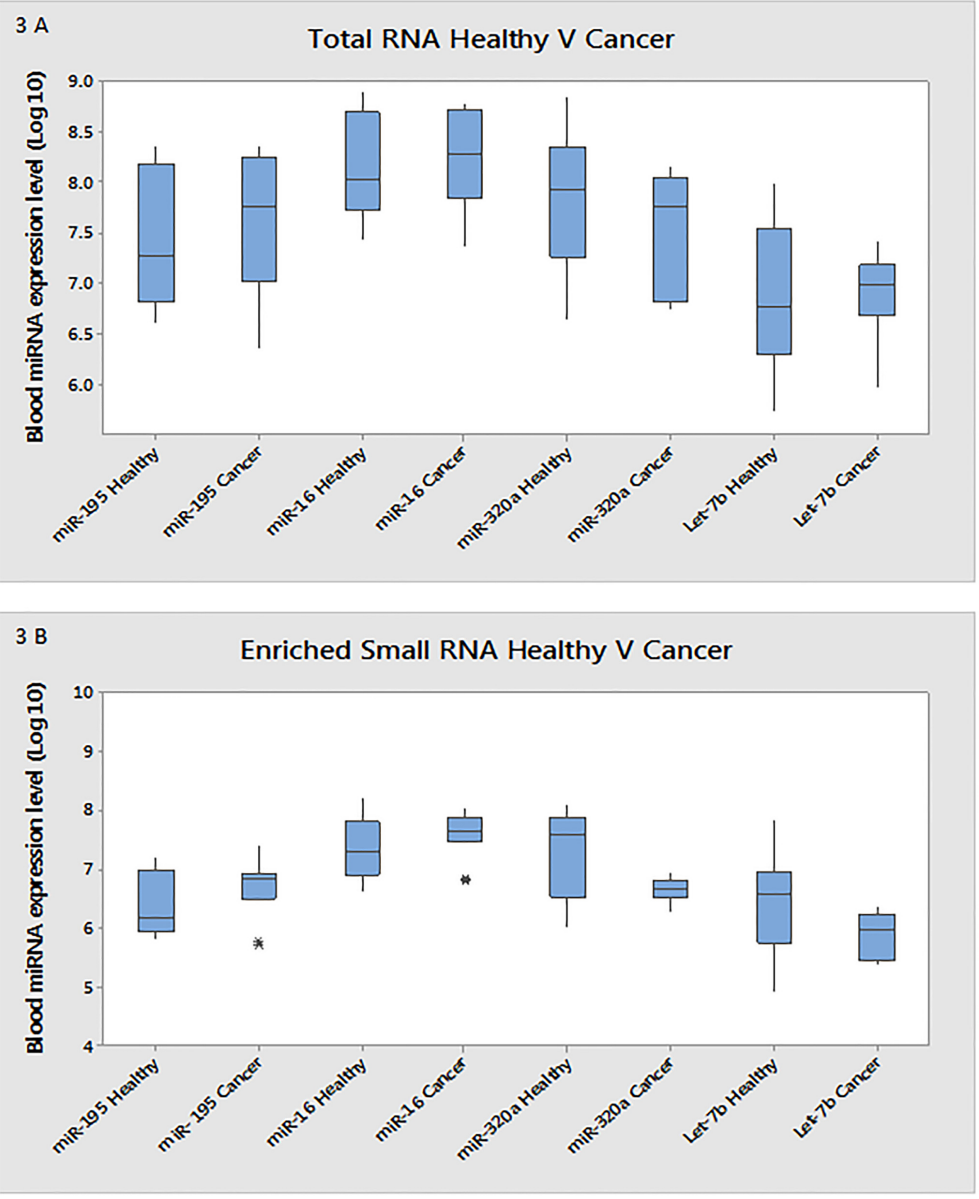
Differential analysis of miRNA isolated from total and enriched RNA from initial sample set. (A) Differential expression of miRNAs between healthy and cancer samples measured in Total RNA from the initial sample set measured using RT-qPCR A 2.3 fold decrease is observed (with a P value of 0.008), in miR-320a from Healthy (1.53E +08) to Cancer (6.63E +07) state. Log 10 box-plots indicating changes in expression of miR-195, miR-16 and Let-7b from healthy to cancer state measured in total RNA using RT-qPCR. (B) Differential expression of miRNAs between healthy and cancer samples measured in enriched small RNA from the initial sample set measured using RT-qPCR. A 5.45 fold decrease is observed (with a P value of 0.0001), in miR-320a from Healthy (4.24E +07) to Cancer (7.78E +06) state. Log 10 box-plots indicating changes in expression of miR-195, miR-16 and Let-7b from healthy to cancer state measured in total RNA using RT-qPCR. *Indicates an outlying value.

An initial validation study was carried out on the RNA isolated from a distinct set of blood samples from a cohort of healthy controls and breast cancer patients. This indicated that the trends observed in the analysis of the small RNA sequencing data were supported in the RT-qPCR analysis of a new sample set. The miR-320a total RNA had a decrease in expression of 2.3-fold (p = 0.008) between the breast cancer samples and healthy control group (Table 5, Fig 3A). Let-7b also demonstrated a two-fold decrease in expression levels from breast cancer patients and the healthy control group (p = 0.03). MiR-195 and miR-16 showed no notable differences between groups (Table 5, Fig 3A).

**Table 5:** Analysis of miRNA dysregulation between healthy and cancer patient samples from the initial sample set.

Total RNA initial sample set					
Two sample T test	Mean	Stdev	SE Mean	P-Value	Fold change Healthy to Cancer
miR-195 Healthy	6.86E+07	8.53E+07	1.38E+07	0.316	1.36
miR-195 Cancer	9.48E+07	8.36E+07	2.16E+07		
miR-16 Healthy	2.51E+08	2.70E+08	4.16E+07	0.933	1.02
miR-16 Cancer	2.56E+08	2.12E+08	5.01E+07		
miR-320a Healthy	1.53E+08	1.83E+08	2.86E+07	0.008	-2.3
miR-320a Cancer	6.63E+07	5.49E+07	1.29E+07		
Let-7b Healthy	2.22E+07	2.93E+07	4.63E+06	0.03	-2.00
Let-7b Cancer	1.11E+07	8.21E+06	1.94E+06		
Enriched small RNA initial sample set					
Two sample T test	Mean	Stdev	SE Mean	P-Value	Fold change Healthy to Cancer
miR-195 Healthy	4.92E+06	5.36E+06	8.69E+05	0.139	1.65
miR-195 Cancer	8.13E+06	7.36E+06	1.90E+06		
miR-16 Healthy	4.76E+07	5.36E+07	8.37E+06	0.729	1.09
miR-16 Cancer	5.18E+07	3.46E+07	8.66E+06		
miR-320a Healthy	4.24E+07	3.84E+07	6.00E+06	0.0001	-5.45
miR-320a Cancer	7.78E+06	1.24E+07	3.10E+06		
Let-7b Healthy	9.15E+06	1.61E+07	2.55E+06	0.003	-8.50
Let-7b Cancer	1.08E+06	7.61E+05	2.03E+05		

**Fig 4:**
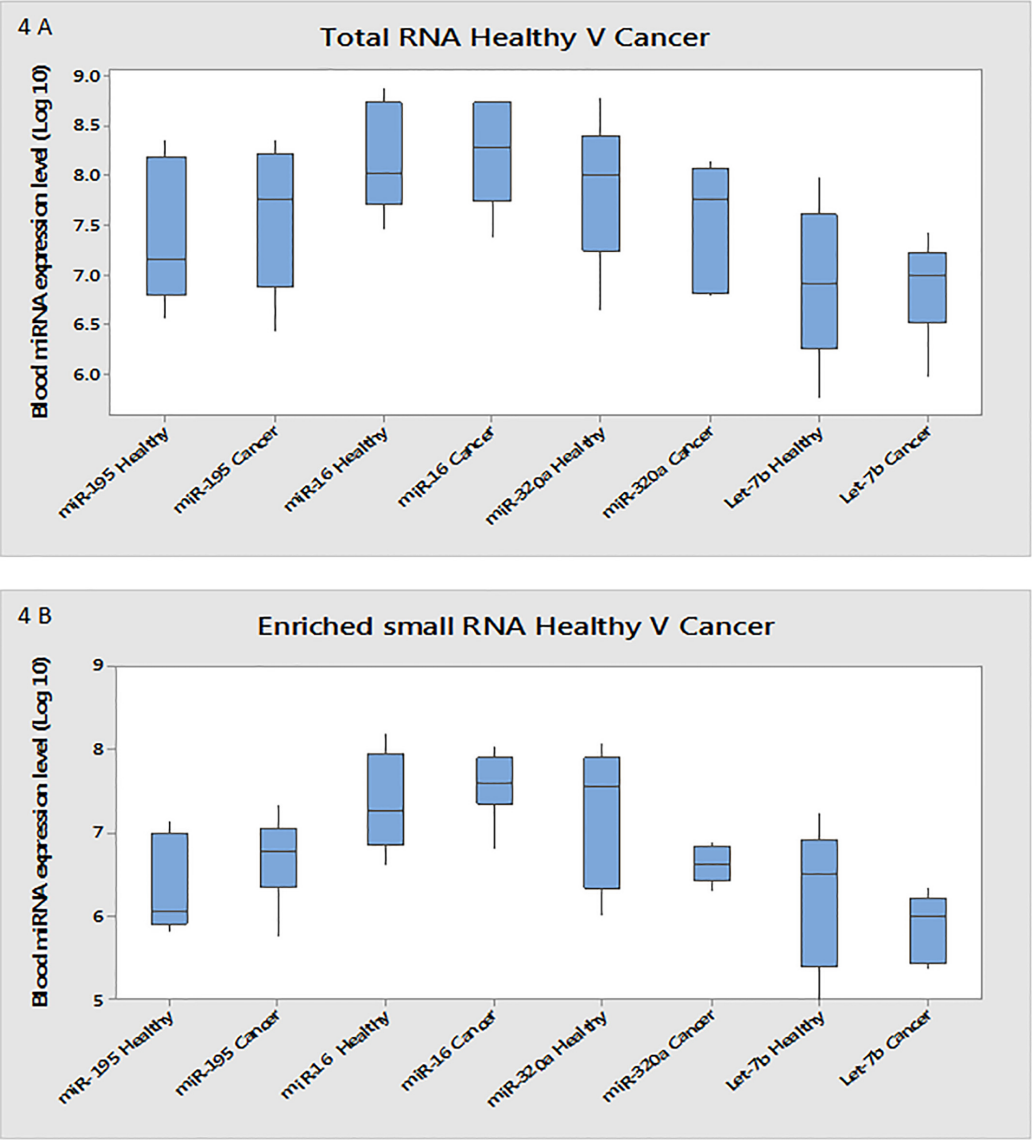
Differential analysis of miRNA isolated from total and enriched RNA from complete sample set. (A) Differential expression of miRNAs between healthy and cancer samples measured in total RNA from the complete sample set measured using RT-qPCR A 2.38 fold decrease is observed (with a P value of 0.111), in miR-320a from Healthy (1.58E +08) to Cancer (6.63E +07) state. Log 10 box-plots indicating changes in expression of miR-195, miR-16 and Let-7b from healthy to cancer state measured in total RNA using RT-qPCR. (B) Differential expression of selected miRNAs between healthy and cancer samples measured in enriched small RNA from the complete sample set measured using RT-qPCR An 8.89 fold decrease is observed (with a P value of 0.006), in miR-320a from Healthy (4.20E +07) to Cancer (4.72E +06) state. Log 10 box-plots indicating changes in expression of miR-195, miR-16 and Let-7b from healthy to cancer state measured in enriched small RNA using RT-qPCR.

The same miRNAs were analysed using enriched small RNA from the same sample set: MiR-320a had 5.45-fold (p<0.0001) and Let-7b 8.5-fold (p = 0.003) lower expression levels in the breast cancer blood samples (Table 5, Fig 3B). No differences in expression levels were found for the miR-195 or miR-16 (Table 5, Fig 3B). MiR-320a was the most dysregulated miRNA across all methods applied here: small RNA sequencing, RT-qPCR of total RNA and RT-qPCR of enriched RNA. A further RT-qPCR analysis of miR-320a was carried out on the total and small RNA from the original samples amplified by sequencing. This was combined with the initial data to create an expanded sample set (n = 55). The miR-320a expression in total RNA across the expanded sample set showed lower expression in the breast cancer blood samples (2.38-fold, p = 0.111) (Table 6, Fig 4A). On enrichment for the small RNA fraction, an 8.89-fold difference (p = 0.006) between the healthy control group and breast cancer patients was observed (Table 6, Fig 4B).

**Table 6:** Analysis of miRNA dysregulation between healthy and cancer patient samples for the complete sample set.

Total RNA full sample set					
Two-sample T-test	Mean	Stdev	SE Mean	P-Value	Fold change Healthy to Cancer
Total miR-320a Healthy	1.58E+08	1.83E+08	4.90E+07		
Total miR-320a Cancer	6.63E+07	5.84E+07	2.38E+07	0.111	-2.38
Enriched small RNA full sample set					
Two-sample T-test	Mean	Stdev	SE Mean	P-Value	Fold change Healthy to Cancer
Enriched Small miR-320a Healthy	4.20E+07	4.03E+07	1.12E+07		
Enriched Small miR-320a Cancer	4.72E+06	2.20E+06	9.83E+05	0.006	- 8.89

## Discussion

The quality and quantity of miRNA isolated from peripheral blood samples is dependent on the total RNA extraction and purification methods used. RNA purification methods have a significant impact on experiment reproducibility [56,57] and this can be compounded by differential RNA degradation rates [58], which can subsequently affect small RNA analysis [32,59]. The PAXgene blood miRNA kit was used for total RNA isolation to maintain consistency as well as reduce bias for both small RNA sequencing and RT-qPCR analysis. Expression profiles generated by this collection and purification system are considered accurate [60,61] and it performs well in comparison to other isolation kits and methods [62,63]. This system also allows the samples to be stored in a frozen state for up to two years without a change in the expression profile [61]. In this study, high quality RNA was recovered from all blood samples, showing RIN of 8 and above. A RIN >5 and preferably >8 is considered ideal for small RNA sequencing and RT-qPCR [64].

High quality RNA is essential for small RNA sequencing, and similarly robust methods for processing are also crucial. The small RNA sequencing normalisation methods used here varied in their detection power due to differences in their scaling and ranking algorithms, particularly in relation to assumptions about extreme value and measurement errors [46–48]. This is caused by low miRNA counts and is an ongoing limitation for RNAseq experiment reproducibility [65]. RPKM rescales as a fraction of the total output, but makes no change to the underlying distribution of expression levels. Quantile normalisation assumes errors are global and affects all genes proportionally [66]. TMM reduces the effects of extreme values and normalises genes with comparable expression levels [67]. By comparing RPKM, upper quantile and TMM approaches, we found that these assumptions about the range and distribution of small RNAs expression levels yielded differing numbers of differentially expressed biomarkers. All three methods in our analysis were applied to maximise specificity at a potential loss of sensitivity. Although differential expression testing power and discovery of novel small RNAs would be improved by increasing sequencing depth and provide a larger sample of small RNAs, the alternative of increasing sample size is more pressing because it would have a larger effect on the biological relevance of the observed changes in expression.

After small RNA sequencing normalisation methods were applied, profiling of whole peripheral blood from the healthy control group and breast cancer patients found evidence of lower miR-320a expression in the latter group, consistent with previous observations [68,69]. MiR-320a is a known tumour-suppressor implicated in several cancers including colon. [70–72] and oral squamous cancer [73]. Additionally, it can bind fatty acid synthase and thus limit the spread of some bone cancers [74]. Other recent work suggests that miR-320a expression may exhibit complex effects dependent on the genetic composition of the breast cancer subtype, but that its dysregulation is linked to poorer survival outcome [75] as supported consistently across all reported investigations, irrespective of methodology the used.

MiR-140 was also identified as dysregulated in the breast cancer blood samples with a 1.72-fold lower expression based on our sequencing data. However, the signal was inconsistent across samples, indicating that it may have diagnostic value in a subset of breast cancer cases. Previously, miR-140 down-regulation was linked to the promotion of cancer stem cell formation in basal-like early stage breast cancer [76]. MiR-140 has also been found to suppress both the metastasis as well as the growth of tumours in patients with non-small cell lung cancer by targeting the insulin-like growth factor 1 receptor [77]. This supports the proposition that targeting miR-140, its regulators, or mRNA targets could improve survival outcomes by regulating stem cell formation [73].

Prior to small RNA sequencing, all samples were processed and enriched for such that only mature miRNAs sequences were examined. To correlate the results obtained from small RNA sequencing and RT-qPCR, the use of a small RNA enrichment step was investigated in this study. A synthetic oligonucleotide representing the precursor to miR-16 was generated for use as control template in a miR-16 Taqman assay. This synthetic precursor oligonucleotide template was detected by the mature miR-16 RT-qPCR assay, indicating a lack of specificity for the mature miRNA (Fig 2, S2 Table). In order to remove this potential for inaccuracy of quantification of miRNAs using qPCR it may also be necessary to employ a size selection step during RNA purification.

As such, for the purposes of this study miR-320a, let-7b, miR-16 and miR-195 were examined by RT-qPCR using both total RNA and also by developing a pre-RT-qPCR enrichment process for RNA <30 bases. Size selection enrichment for the small RNA population decreased RNA copy number, but may have increased the fraction of mature RNAs (Table 4). The decrease in copy numbers of specific miRNAs observed from total RNA to enriched small RNA samples highlights the potential for error in quantification due to precursor miRNAs in the total RNA. A non-human miRNA (*Arabidopsis* miR-159) was measured before and after small RNA enrichment as a control. This miRNA had a consistent Cq of 27±3 in total RNA and 31±5 in enriched small RNA. The RT-qPCR data was normalised using the median miR-159 expression to reduce potential bias. The data generated for the enriched small RNA consistently showed less variance and were more reliable than those for the total RNA. The steps taken in processing the samples and the data analysis have resulted in an increased accuracy in the measurement of mature miRNA targets and thus superior inference power due to lower variation in expression due to experimental procedures.

These standardisation steps facilitated the validation by RT-qPCR relative expression differences first observed in small RNA sequence reads. The size selection step and miR-159 control normalisation indicated an 8.89-fold down-regulation of miR-320a in circulating blood from breast cancer patients relative to the healthy control group. Let-7b also had lower expression in circulating blood from the breast cancer cohort, an observation that was not statistically supported by sequencing results, and no evidence of differential expression was observed using either method for miR-195 or miR-16. This finding is consistent with the use of miR-16 as a stable endogenous control [78].

These findings serve to demonstrate the utility of the standardisation and normalisation steps carried out in the discovery and initial validation of potential biomarkers for disease through the comparison of data across platform types. This study developed a suitable RT-qPCR miRNA quantification protocol that produced measurements comparable with small RNA sequencing output. This supports the perspective that relative gene expression levels can be measured in a reproducible manner across platforms [79], so that high-throughput miRNA discovery in pilot cohorts can be validated in larger patient groups using RT-qPCR. Imprecision in RT-qPCR protocols and primer sequence design can yield inaccurate measurements as can GC bias in small RNA sequencing [80] or in TRIzol extraction [81], and due to local sequence artefacts [82]. The reduction of experimental variation in the process of miRNA quantification from sample collection to final interpretation through the use of the steps outlined in this study allow for more robust and comparable findings. By applying these methods to whole peripheral blood RNA from healthy control group and breast cancer patients, miR-320a expression was down regulated using both small RNA sequencing and RT-qPCR. However, the clinical significance of this miRNA as a potential biomarker for breast cancer from circulating blood must now be evaluated further using larger cohort sample sizes.

## Conclusion

The advances in the field of circulating miRNAs and their relevance for disease profiling and treatment have dramatically increased interest in their utility as biomarkers. They exhibit many features that make them ideally suited to use as diagnostic and prognostic biomarkers. The lack of consistency in reporting and the difficulty comparing data from one study to another are major stumbling blocks in the advancement of miRNAs as clinically relevant biomarkers [83].

On commencement of this study there was an absence of a standardised protocol for the treatment of RNA samples from isolation from the blood to expression comparison between methods. In order to facilitate cross study comparison of data and to increase the accuracy, robustness and repeatability of findings, the development of such a protocol was important. Overall, our findings emphasise the importance of the use of a standardised protocol in the processing of RNA samples and the development of miRNA profiles.

We have demonstrated an initial proof of concept for a pipeline of controlled steps involving standardisation and normalisation that serves to reduce the potential for bias, inaccuracy and variability in the findings. Through further validation of this pipeline and its application in cross platform studies, it will be possible to accurately compare miRNA profiles both between small RNA sequencing and RT-qPCR. If miRNA quantification and profiling studies used a standardised approach, it may become possible to accurately compare data generated in separate studies. This would decrease the potential for conflicting results and provide a global resource of data that would lead to an enhanced potential for meaningful developments in the field of miRNA profiling.

## Supporting Information

S1 Fig Global miRNA expression profiles of (A) control and (B) breast cancer patient samples. These profiles are comparable with no major differences between patient cohorts. The most highly expressed miRNA (miR-451a) accounted for 60% (control) and 65% (breast cancer) of the total miRNA population.
(PDF)

S2 Fig Global unannotated small RNA expression profiles of (A) control and (B) breast cancer patient samples. These profiles are comparable with no major differences between cohorts. The percentages correspond to values across all small RNAs only, which accounted for 2.5% (control) and 2.6% (breast cancer) of the total small RNA population. The ten small RNAs shown account for 48.5% (control) and 45.2% (breast cancer) of the total. ENST00000364228 was the most highly expressed in both groups (17.2% of total in controls, 14.0% of total in breast cancer cohort).
(PDF)

S3 Fig Representative standard curve employed for Aly-miR-159 spike-in control assay. This spike-in control assay was used for normalisation of RT-qPCR data.
(PDF)

S1 Table Small unannotated RNAs detected unique to breast cancer patients. (PDF)

S2 Table Cq values obtained for mature and precursor miR-16 assay. (PDF)
