# Supplementary material for: Cross Platform Standardisation of an Experimental Pipeline for Use in the Identification of Dysregulated Human Circulating MiRNAs
Source: PLoS One. 2015 Sep 10;10(9):e0137389. doi: 10.1371/journal.pone.0137389 (PMC4565682; doi:10.1371/journal.pone.0137389)
Supplement: S1 Fig — These profiles are comparable with no major differences between patient cohorts. The most highly expressed miRNA (miR-451a) accounted for 60% (control) and 65% (breast cancer) of the total miRNA population. (PDF) [file pone.0137389.s001.pdf]

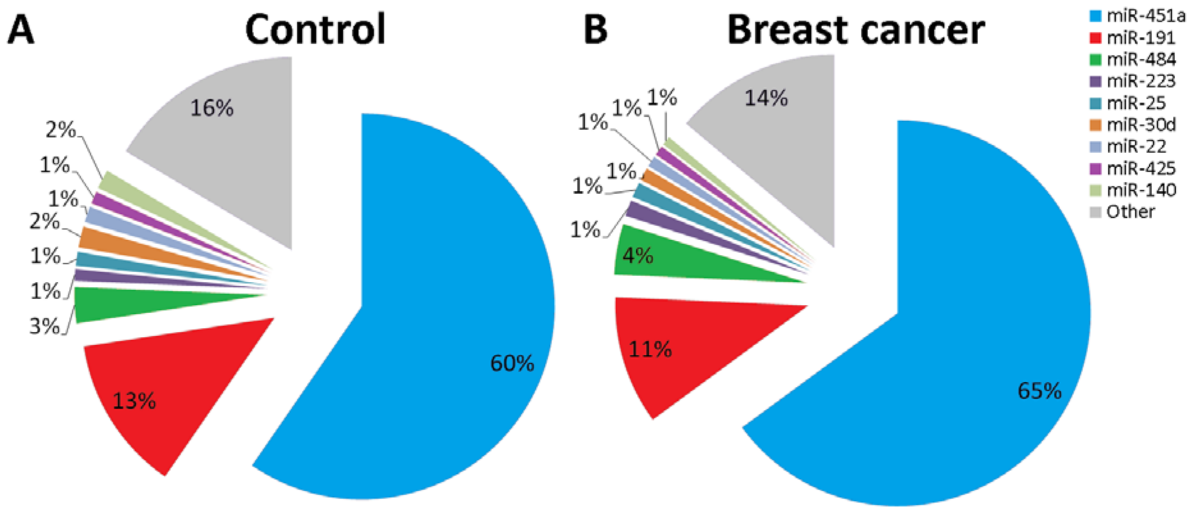

S1 Fig: Global miRNA expression profiles of (A) control and (B) breast cancer patient samples. These profiles are comparable with no major differences between patient cohorts. The most highly expressed miRNA (miR-451a) accounted for 60% (control) and 65% (breast cancer) of the total miRNA population.
