# Supplementary material for: Cross Platform Standardisation of an Experimental Pipeline for Use in the Identification of Dysregulated Human Circulating MiRNAs
Source: PLoS One. 2015 Sep 10;10(9):e0137389. doi: 10.1371/journal.pone.0137389 (PMC4565682; doi:10.1371/journal.pone.0137389)
Supplement: S2 Fig — These profiles are comparable with no major differences between cohorts. The percentages correspond to values across all small RNAs only, which accounted for 2.5% (control) and 2.6% (breast cancer) of the total small RNA population. The ten small RNAs shown account for 48.5% (control) and 45.2% (breast cancer) of the total. ENST00000364228 was the most highly expressed in both groups (17.2% of total in controls, 14.0% of total in breast cancer cohort). (PDF) [file pone.0137389.s002.pdf]

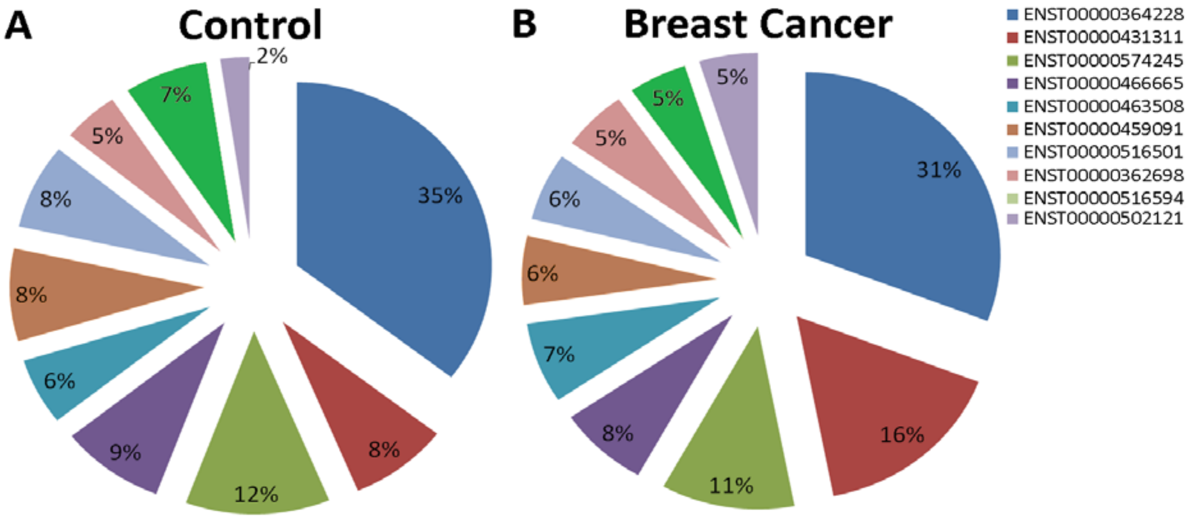

S2 Fig: Global unannotated small RNA expression profiles of (A) control and (B) breast cancer patient samples. These profiles are comparable with no major differences between cohorts. The percentages correspond to values across all small RNAs only, which accounted for 2.5% (control) and 2.6% (breast cancer) of the total small RNA population. The ten small RNAs shown account for 48.5% (control) and 45.2% (breast cancer) of the total. ENST00000364228 was the most highly expressed in both groups (17.2% of total in controls, 14.0% of total in breast cancer cohort).
