# Supplementary material for: Cross Platform Standardisation of an Experimental Pipeline for Use in the Identification of Dysregulated Human Circulating MiRNAs
Source: PLoS One. 2015 Sep 10;10(9):e0137389. doi: 10.1371/journal.pone.0137389 (PMC4565682; doi:10.1371/journal.pone.0137389)
Supplement: S3 Fig — This spike-in control assay was used for normalisation of RT-qPCR data. (PDF) [file pone.0137389.s003.pdf]

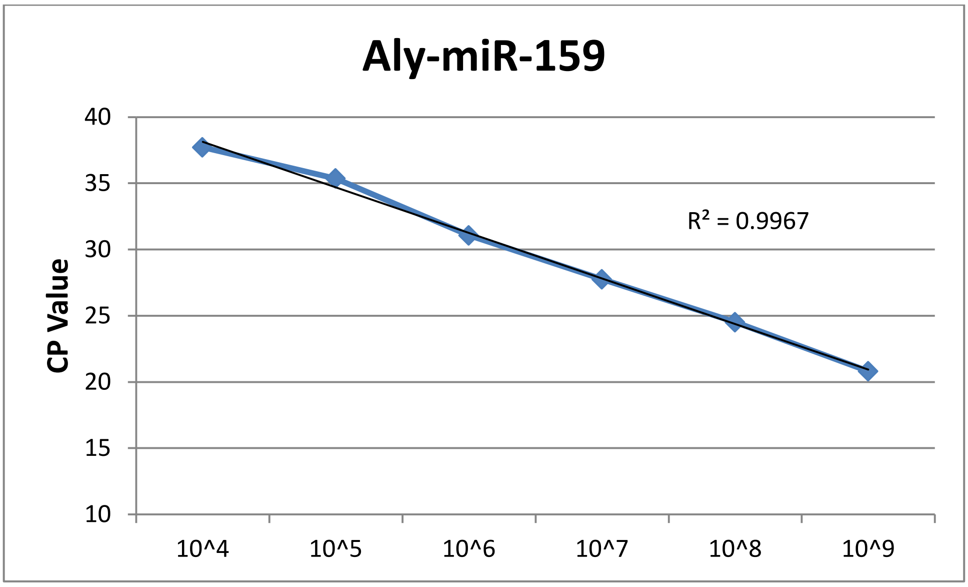

S3 Fig: Representative standard curve employed for Aly-miR-159 spike-in control assay. This spike in-control assay was used for normalisation of RT-qPCR data.
