# Supplementary material for: Cross Platform Standardisation of an Experimental Pipeline for Use in the Identification of Dysregulated Human Circulating MiRNAs
Source: PLoS One. 2015 Sep 10;10(9):e0137389. doi: 10.1371/journal.pone.0137389 (PMC4565682; doi:10.1371/journal.pone.0137389)
Supplement: S1 Table — (PDF) [file pone.0137389.s004.pdf]

S1 Table: Small unannotated RNAs detected unique to breast cancer patients.

Unannotated small RNA	Mean	Total
ENST00000490626	14.1	211
ENST00000514339	6.3	94
ENST00000447709	4.2	63
ENST00000420721	3.9	58
ENST00000362585	3.9	59

The five listed had expression levels greater than the mean total counts per gene in the cancer cohort (57.9).

ENST00000490626 was detected most frequently in the breast cancer cohort (7 out of 15)
