# Supplementary material for: Cross Platform Standardisation of an Experimental Pipeline for Use in the Identification of Dysregulated Human Circulating MiRNAs
Source: PLoS One. 2015 Sep 10;10(9):e0137389. doi: 10.1371/journal.pone.0137389 (PMC4565682; doi:10.1371/journal.pone.0137389)
Supplement: S2 Table — (PDF) [file pone.0137389.s005.pdf]

S2 Table: Cq values obtained for mature and precursor miR-16 assay

Sample	concentration	Cq	Concentration
miR-16	10^8	23.40	1.00E+08
miR-16	10^7	26.83	1.00E+07
miR-16	10^6	30.37	7.94E+05
miR-16	10^5	32.56	1.40E+05
miR-16	10^4	35.75	9.02E+03
NTC	Negative	-ve	-ve
pre-miR-16	10^8	28.62	2.88E+06
pre-miR-16	10^7	31.80	2.58E+05
pre-miR-16	10^6	33.64	5.69E+04
